# Extracellular and Intracellular Concentrations of Molybdenum and Zinc in Soccer Players: Sex Differences

**DOI:** 10.3390/biology11121710

**Published:** 2022-11-25

**Authors:** Víctor Toro-Román, María Concepción Robles-Gil, Diego Muñoz, Ignacio Bartolomé, Jesús Siquier-Coll, Marcos Maynar-Mariño

**Affiliations:** 1 Faculty of Sport Sciences, University of Extremadura, Avenida de la Universidad s/n, 10003 Cáceres, Spain; 2 Department of Sport Science, Faculty of Education, Pontifical University of Salamanca, C/Henry Collet, 52-70, 37007 Salamanca, Spain; 3 SER Research Group, Center of Higher Education Alberta Giménez, Comillas Pontifical University, Costa de Saragossa 16, 07013 Palma Mallorca, Spain

**Keywords:** plasma, urine, erythrocytes, platelets, male, female

## Abstract

**Simple Summary:**

Zinc (Zn) and molybdenum (Mo) are trace minerals elements (TMEs) found in plant and animal foods. TMEs play an important role in various metabolic functions of the body. Regulation of TMEs in blood and tissues is important for these functions both at rest and during physical exercise and it is known that physical exercise can cause a redistribution of TMEs between body stores, blood and tissues. This study analyzed sex differences in plasma, urine, erythrocytes and platelets concentrations of Zn and Mo in soccer players and related these concentrations to biochemical parameters of muscle damage. The present study involved 68 male and 70 female soccer players. Erythrocytes, platelets, creatine kinase (CK), and lactate dehydrogenase (LDH) values were also determined. In addition, nutritional intake, body composition, and physical condition were assessed. Men soccer players obtained higher plasma and urinary concentrations of Mo and Zn. Women soccer players showed higher Zn concentrations in erythrocytes. Extracellular concentrations of Mo and Zn were higher in male soccer players. However, the women soccer players showed higher erythrocyte Zn concentrations.

**Abstract:**

Molybdenum (Mo) and zinc (Zn) play important roles in the process of adaptation to physical training. The aims of the present study were: (i) to analyze the differences in extracellular (plasma and urine) and intracellular (erythrocytes and platelets) Mo and Zn concentrations between sexes and (ii) to relate extracellular Zn concentrations with biomarkers of muscle damage and muscle mass. The present study involved 138 semi-professional soccer players divided according to sex: male (n = 68) and female (n = 70). Mo and Zn concentrations were determined by inductively coupled plasma mass spectrometry. Erythrocytes, platelets, creatine kinase (CK), and lactate dehydrogenase (LDH) values were also determined by automatic cell counter and spectrophotometric techniques. There were no sex differences in Mo and Zn intake. Male soccer players obtained higher values of erythrocytes, CK, and LDH (*p* < 0.05), and showed higher plasma and urinary concentrations of Mo and Zn (*p* < 0.05). Female soccer players showed relatively higher Zn concentrations in erythrocytes (*p* < 0.05). Finally, positive correlations were observed between extracellular Zn concentrations with CK, LDH and muscle mass. Extracellular concentrations of Mo and Zn were higher in male soccer players. However, the relative concentrations of Zn in relation to the number of erythrocytes were higher in female soccer players.

## 1. Introduction

Trace mineral elements (TMEs) are inorganic micronutrients found in plant and animal foods [1]. TMEs play important roles in various metabolic functions of the body [2]. The regulation of TMEs in blood and tissues is important for these functions both at rest and during physical exercise [3], and it is known that physical exercise can cause a redistribution of TMEs among body stores, blood, and tissues [4]. In addition, TME status can affect performance in athletes [5,6]. Concerning the above, some TMEs such as molybdenum (Mo) and zinc (Zn) are important to generate adaptative processes to physical training [7,8].

Mo presents biological activity when it binds to a cofactor. It is abundant in the sea and is predominantly found in the form of the oxyanion molybdate [9]. More than 50 Mo enzymes have been described in nature [10]. In humans, four molybdoenzymes have been identified [1,11]: (i) aldehyde oxidase, whose function is to oxidize and detoxify various pyrimidines and purines; (ii) xanthine oxidase/dehydrogenase, responsible for catalyzing the transformation of hypoxanthine to xanthine and xanthine to uric acid; (iii) sulfite oxidase, whose function is to catalyze the transformation of sulfite to sulfate; and (iv) mitochondrial amidoxime reducing component (mARC), which was recently discovered and plays a role in the detoxification of N-hydroxylated substrates. Being a transition element, Mo acts as an electron transfer agent in oxidation–reduction reactions [12].

Zn is one of the most studied TMEs due to its abundance in organisms [13]. Zn is a cofactor for more than 300 metalloenzymes and is involved in different biological functions: immunity, energy metabolism or the antioxidant system, among others [14]. Zn concentrations are found predominantly in skeletal muscle (60%) and bone (30%) [13]. Intramuscular Zn concentrations provide structural integrity and contribute to the enzymatic activities of metalloenzymes, such as lactate dehydrogenase (LDH) and carbonic anhydrase (CA) [15]. 

Normally, Mo and Zn status is determined by analyzing plasma or serum concentrations [16,17,18]. However, it does not seem to be a specific indicator of Zn status in athletes or the general population [19,20], with no information available for Mo. In view of the above, previous authors proposed simultaneously assessing TME status in different compartments [4,13]. 

Although Mo and Zn concentrations are usually under homeostatic control, physical exercise induces alterations. Since the musculoskeletal system contributes the highest percentage of total body Zn, it has been proposed that physical exercise and subsequent recovery processes influence Zn homeostasis [18]. In this regard, it has been observed that serum Mo and Zn concentrations differ among sports modalities and training levels [21,22], as well as erythrocyte concentrations [7]. In soccer players, Algul et al. [23] reported that plasma Zn concentrations decreased at the end of a match, whether the game was played in the morning or in the evening. Metin et al. [24] observed no differences in Zn concentrations between male and sedentary soccer players. However, Toro-Román et al. [13] reported higher plasma concentrations and lower erythrocyte concentrations of Zn in soccer players compared to a control group. 

Few investigations have analyzed sex differences in Zn concentrations. In this regard, Lukaski et al. [25] reported higher plasma Zn concentrations in male swimmers compared to female swimmers. On the other hand, Haralambie et al. [26] showed in 160 athletes (57 women) that 23.3% of the male athletes and 43% of the female athletes examined presented hypozincemia (<75 pg/dL serum Zn). Regarding Mo, no information was found.

The sex difference in muscle mass could influence extracellular Zn concentrations [27] since an important part of Zn is found in this tissue, and the muscle damage produced by physical exercise could favor the release of Zn from muscle tissue to extracellular compartments [13]. Likewise, the number of erythrocytes could affect intracellular Zn concentrations because Zn acts as a cofactor of CA and superoxide dismutase (SOD), also present in the erythrocyte membrane [28]. Regarding sex differences in Mo concentrations, as discussed above, no data have been reported. 

Therefore, due to the scarce information on sex differences in Mo and Zn concentrations, the objectives of the present study were: (i) to analyze the differences in extracellular (plasma and urine) and intracellular (erythrocytes and platelets) Mo and Zn concentrations between sexes and (ii) to relate extracellular Zn concentrations to biochemical parameters of muscle damage and muscle mass.

## 2. Materials and Methods

### 2.1. Study Design

**Figure 1:**
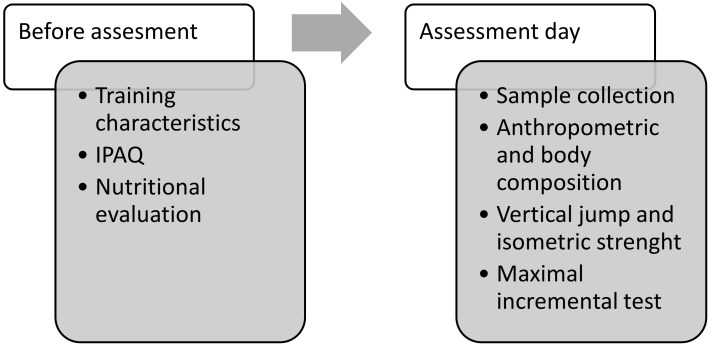
Study design.

In the present quasi-experimental cross-sectional study, the assessments were performed between the second and third week after the start of the season in the following order: nutritional analysis, physical activity questionnaire, blood extraction and urine collection, anthropometry, vertical jump, isometric strength of the lower limb and maximal incremental test. The protocol was approved by the Biomedical Ethics Committee of the University of Extremadura (Spain), following the Helsinki Declaration of ethical guidelines for research on human subjects (135/2020). Figure 1 describes the study design and the order of the assessments conducted.

### 2.2. Subjects

To participate in the study, subjects had to meet the following criteria: (a) not to smoke; (b) not to drink alcohol during 10 days before the assessments; (c) to have at least 5 years’ experience competing in soccer; (d) not to have followed any special diet in the previous 3 months; (e) not to have taken any multivitamin/mineral supplements in the previous 3 months; (f) not to have suffered any type of illness or injury that had prevented the player from training in the previous 3 months; and (g) to have resided in the city of Cáceres (Spain) for at least 2 years. In addition to the above criteria, the female participants had to meet the following additional criteria: (a) to have regular menstrual cycles during the 6 months before the study; (b) not to suffer from problems related to the menstrual cycle and (c) not to use contraceptive methods. 

Based on the above criteria, a total of 138 soccer players participated in the present study. The participants were divided according to sex into male soccer players (n = 68) and female soccer players (n = 70). The male soccer players belonged to semi-professional senior teams in the third and fourth categories of Spanish soccer. The female soccer players belonged to semi-professional clubs playing in the second and third categories of Spanish women’s soccer. Both clubs trained and played matches in Cáceres (Spain). All participants signed a consent form prior to enrolment.

The training characteristics of the participants were obtained from the data provided by the coaching staff of the different teams (Table 1). Moreover, the subjects completed the physical activity questionnaire—short form (IPAQ-SF) Spanish version to record their levels of physical activity [29,30].

### 2.3. Nutritional Evaluation

The participants completed a nutritional questionnaire to ascertain the intake of Mo and Zn. The nutritional composition of each food was assessed using food composition tables [31,32]. A database was created from the above tables. Participants were provided with a document where they had to indicate the amount and frequency of food intake in the 3 days before the assessments (2 working days and 1 weekend day). The researchers performed conversion of proportion to estimate the consumption of each micronutrient using the above food tables and assisted participants during data collection. Once the documents were collected from all participants, the amount of macronutrients, Mo, Mn, and water were calculated concerning the database using the food composition tables. The average of the three days was taken into account for the statistical analysis. For greater precision, a support document was sent to estimate the quantities of each food ingested according to the container used in order to reduce measurement bias.

### 2.4. Anthropometric and Body Composition

Anthropometric assessments were performed after fasting blood draws and with as little clothing as possible. A tetracompartmental model was used. Following the guidelines of Porta et al. [33], body composition parameters were obtained, and the same evaluator performed the different anthropometric assessments: height, weight, skinfolds (abdominal, suprascapular, subscapular, tricipital, thigh, and calf), bone diameters (bistyloid, humerus and femur) and muscle perimeters (relaxed arm and calf). A wall-mounted stadiometer (Seca 220, Hamburg, Germany), an electronic digital scale (Seca 769, Hamburg, Germany), a Holtain© 610ND skinfold compass (Holtain, Crymych, UK), a Holtain© 604 pachymeter (Holtain, Crymych, UK), and a Seca© 201 tape measure (Seca, Hamburg, Germany) were used. The Yuhasz equation was used to calculate the fat percentage (1) [34]. The muscle percentage was obtained by dividing the muscle weight, obtained by subtracting the body, bone (Von Doblen equation modified by Rocha [33]), fat and residual (Wurch equation [33]) weights, with the total weight and divided by 100. Three assessments were made for each parameter (skinfolds, bone diameters and muscle perimeters) and the mean was chosen for statistical analysis. The muscle/bone ratio was determined to determine a musculoskeletal index.

Fat (%) = 4.56 + (Σ6 skinfold [mm] × 0.143) (female)

Fat (%) = 3.64 + (Σ6 skinfold [mm] × 0.097) (male)

### 2.5. Vertical Jump and Isometric Strength

The vertical jump squat jump (SJ) test and an isometric test of the lower limbs with a dynamometer were performed to assess lower limb power and strength. Before performing both tests, the following warm-up was performed: (1) knee and hip mobility and (2) 4–5 half squats without load and then isometric squats for 5 s.

SJ was performed following the guidelines of Bosco et al. [35]. A photocell platform (Optojump, Mycrogate, Mahopac, New York, NY, USA) was used. To perform SJ, participants started in a squat position with knees bent at 90° and arms at the hips. A goniometer was used to verify the initial knee angle. Participants were required to remain in this squat position for 3 s and then perform a rapid upward movement. The jump was repeated 2 times and the best one was selected for further analysis. There was 30 s rest between repetitions.

A lower limb isometric strength assessment was performed with a dynamometer (TKK5402, Takei, Tokyo, Japan) after a warm-up similar to the SJ. Participants started the test with knees flexed between 90–100°, back straight, and holding the handle approximately at knee height. In that position, participants executed a maximal isometric upward contraction. The contraction was stopped when the values displayed on the dynamometer screen did not increase. Two attempts were performed and the best one was selected for analysis. There was 2 min of recovery between attempts.

### 2.6. Maximal Incremental Test

The maximal incremental test was performed on a treadmill (Ergofit Trac Alpin 4000, Kübler Sport GmbH, Backnang, Germany), equipped with a gas analyzer (Geratherm Respiratory GMBH, Ergostik, Ref 40.400, Corp., Bad Kissingen, Germany) and a Polar heart rate monitor (Polar^®^ H10, Kempele, Finland). The protocol consisted of running in 1 min stages until exhaustion. The test started at 7 km/h and increased by 1 km/h every minute with a stable gradient of 1%. Prior to the test, a 15 min warm-up on the treadmill was performed where participants ran at 6 km/h. All tests were performed after a free breakfast. To consider the incremental test valid, the following criteria were selected: (a) respiratory coefficient >1.10 and (b) plateau in the heart rate graph.

### 2.7. Sample Collection

The techniques used to obtain the different biological matrices were similar to those reported by Toro-Román et al. [4,13].

Fasting blood samples were taken at 8:00 a.m. In addition, participants came with the first urine of the morning collected. Twelve mL of blood were drawn using a 20 mL plastic syringe (Injekt, Braun, Melgunsen, Germany) and a G21 sterile moth needle (Mirage Pic Solution, Trieste, Italy). Of the total, 2 mL were collected in Vacutainer^®^ tubes with EDTA (ethylenediaminetetraacetic acid) to determine hematological parameters using an automatic cell counter (Coulter Electronics LTD, Model CPA; Northwell Drive, Luton, UK). Similarly, 2 mL of blood were collected to determine the muscle damage markers creatine kinase (CK) and lactate dehydrogenase (LDH) by spectrophotometric techniques (Advia 1650; Bayer, Leverkusen, Germany).

The remaining 8 mL were used to determine the concentrations of Mo and Zn in the different biological matrices. Two 4 mL BD Vacutainer^®^ tubes with sodium citrate were collected. To obtain the plasma, a Vacutainer^®^ tube with metal-free sodium citrate was taken and centrifuged at 1800 rpm for 8 min. The platelet-rich plasma (PRP) obtained was collected in dry BD Vacutainer^®^ tubes without additives and centrifuged for 10 min at 3000 rpm. The plasma was aliquoted into 1.5 mL Eppendorf tubes (previously washed with dilute nitric acid) and allowed to stand at −80 °C until further analysis. 1 mL of pure water (Mili-Q) was added to the adherent platelets and agitated in a vortex (Cole-Parmer™, Stuart™, Vernon Hills, IL, USA) for mixing. The mixture was transferred to an Eppendorf tube and stored at −80 °C.

Erythrocytes were removed from the remaining blood and washed three times with 0.9% sodium chloride (NaCl). They were then aliquoted into 1.5 mL Eppendorf tubes (previously washed with dilute nitric acid) and stored at −80 °C until biochemical analysis.

Urine was collected by participants in 100 mL cups. Urine samples were transferred into 9 mL BD Vacutainer^®^ tubes and frozen at −80 °C until analysis. Prior to analysis, all samples were thawed and homogenized by shaking.

### 2.8. Mo and Zn Determination

The determination of Mo and Zn was similar to that reported in previous investigations [13,21]. The method was developed entirely at the Elemental and Molecular Analysis Service of the Research Support Services of the University of Extremadura (Spain).

The determination of Mo and Zn was performed by inductively coupled plasma mass spectrometry (ICP-MS) (7900; Agilent Tech., Santa Clara, CA, USA). The instrument has a fast dual simultaneous mass detector (ODS), a high-frequency hyperbolic quadrupole and a fourth-generation reaction octopole system that allows operation in two modes: STD (no reaction gas) and KED (kinetic energy discrimination with helium as the collision gas). Both the collision gas and argon for the plasma were 99.999% pure and were supplied by Praxair (Madrid, Spain). In addition, it has a 27 MHz variable frequency generator, cooled spray chamber, low-flow sample introduction system, off-axis ionic lenses and an extraction interface with high transmission and matrix tolerance.

The linearity of the calibration curves for In in plasma, urine, erythrocytes and platelets were greater than 0.985. The values of the standard materials for this element (10 μg/L) used for quality controls coincided with intra- and inter-assay coefficients of variation lower than 5%.

Sample preparation: for plasma and urinary samples, the reagents used were 69% nitric acid (TraceSELECT™, Fluka™, Madrid, Spain) and ultrapure water obtained from a Milli-Q system (Millipore^®^, Burlington, MA, USA). A 400 µg·L^−1^ rhodium dilution was used as the internal standard, which was continuously fed into the apparatus with the help of a three-channel peristaltic pump. 0.2 mL of the samples were added to a volume of 5 mL with a 1% nitric acid solution prepared from a commercial one of 69%. The equipment was calibrated with several calibration standards prepared from commercial multi-elemental solutions of certified standards.

For erythrocyte and platelet samples, the reagents used in method development and sample analysis were 69% nitric acid, hydrogen peroxide (TraceSELECT™, Fluka™, Madrid, Spain), and ultrapure water obtained from a Milli-Q system (Millipore^®^, Burlington, MA, USA). A 400 µg·L^−1^ yttrium and rhodium dilution of 400 µg·L^−1^ was continuously fed into the apparatus with the aid of a three-channel peristaltic pump and was used as an internal standard. 

Samples were weighed on a precision balance of ±0.4 g and introduced into glass tubes for microwave digestion, adding 3.5 mL of a 3:1 mixture of 69% nitric acid and hydrogen peroxide (TraceSELECT™, Fluka™, Madrid, Spain).

Once digested, the resulting solutions were diluted to 25 mL with Milli-Q water. The equipment was calibrated with several calibration standards prepared from commercial multi-elemental solutions of certified standards. The limits of detection and limits of quantification for Mo and Zn were 0.0018/0.18 (µg/L) and 0.034/0.34 (µg/L), respectively.

### 2.9. Statistical Analysis

The Kolmogorov–Smirnov normality test was applied. The homogeneity of the variances was tested with the Levene test. The parametric *t*-test for independent samples was used to observe the differences between male and female players. The results are expressed as means ± standard deviations. Pearson’s correlation coefficient (*r*) was used to determine the relationships between the variables. The level of significance was set at *p* < 0.05. Statistical analyses were performed with IBM SPSS Statistics 22.0.

## 3. Results

Table 2 shows that male players obtained higher values in height, weight, skinfold sum, muscle percentage, bone percentage and muscle/bone ratio, SJ height, SJ flight time, lower limb isometric strength, total time in the maximum incremental test, maximum speed reached in the test, maximum volume of oxygen consumption (VO_2max_) and maximum volume of carbon dioxide output (VCO_2max_) compared to female soccer players (*p* < 0.05). On the other hand, female players showed higher fat percentage (*p* < 0.01) and higher values in SJ height in relation to body weight (*p* < 0.05). 

Table 3 shows that male soccer players consumed more energy (*p* < 0.05).

Table 4 indicates that male players showed higher erythrocyte, CK and LDH values compared to female players (*p* < 0.01). However, female players had higher platelet concentrations (*p* < 0.05).

Table 5 and Table 6 show the concentrations of Mo and Zn. Male soccer players showed higher Mo concentrations in plasma and urine compared to female soccer players (*p* < 0.05). On the other hand, male players presented higher Zn concentrations in plasma and urine, (*p* < 0.01). However, female players showed higher Zn concentrations in erythrocytes in relative values (*p* < 0.01).

Table 7 reports that positive correlations were observed between plasma Zn, muscle percentage and muscle/bone ratio (*p* < 0.05). Regarding urinary Zn, there were positive correlations with the biomarkers of muscle damage, muscle percentage, muscle/bone ratio (*p* < 0.05).

No significant correlations were observed in Table 8. However, all the relationships were positive.

## 4. Discussion

The present investigation aimed to analyze sex differences in Mo and Zn concentrations in different biological matrices and to relate extracellular Zn concentrations to markers of muscle damage and muscle mass. Male soccer players showed higher plasma and urine concentrations of Mo and Zn. However, they showed lower Zn concentrations in relative values in erythrocytes compared to female soccer players. Regarding the correlations between extracellular Zn concentrations with biomarkers of muscle damage and muscle percentage, when analyzing the participants in general, significant correlations were observed, especially for urinary Zn concentrations. When dividing between sexes, positive correlations were observed without being significant. The present investigation resolves some of the limitations raised in previous work on Zn [13]. Among them, direct relationships between markers of muscle damage and extracellular Zn concentrations. Muscle damage could induce an increased release of Zn from muscle cells into plasma, as most Zn concentrations predominate mainly in skeletal muscle [15,36]. The present investigation provides new information on sex differences in the various Zn and Mo concentrations in athletes. Likewise, it provides data on Mo concentrations in female athletes. All values obtained in the present study were within a range reported in previous investigations [37,38]. 

Different markers have been used to assess Mo and Zn status. Plasma and serum Mo concentrations are low in humans and therefore complex to assess. Consequently, there are few studies on Mo concentrations in these biological compartments [39]. Moreover, the main route of Mo excretion is urine [40]. Urinary Mo concentrations reflect dietary intake, with concentrations increasing as dietary intake increases. However, urinary Mo alone does not reflect Mo status [39]. Regarding Zn, biological compartments such as plasma, serum, erythrocytes or urine have been used to assess its status [41], in addition to specific enzymes and proteins. However, there is no consensus on which indicators are appropriate for determining Zn status. This has led to various indicators being used, making comparison among investigations difficult [42]. Although serum Zn concentration is the most commonly used marker to determine Zn status, factors such as age, hormones, stress, infection, and inflammation can affect these concentrations [17,20]. Therefore, in view of the above, it is necessary to consider other compartments to assess Zn status. Assessments of intracellular Zn concentration is not common in the scientific literature [43]. The assessment of Zn in erythrocytes could be accepted as it does not seem to be influenced by acute inflammatory responses or diet in the short term [44]. Due to the drawbacks of some biological matrices, it is important when assessing TME states to determine the extracellular and intracellular concentrations in different compartments simultaneously. Concerning the above, the present study provides information on platelet concentrations of Zn and Mo in female athletes. 

Both groups’ intakes of Mo and Zn were similar and higher than dietary reference intakes (45 μg/day Mo; 9.5 mg/day of Zn in males and 7 mg/day of Zn in females) [45]. Mo intakes were higher than the average reported in the United States, Korea, and Japan, where rice consumption contributes to a higher Mo intake [11]. Cereals, including wheat, oats, and rice, are a major source of Mo. A large percentage of the nutritional intake of soccer players corresponds to carbohydrates [46], which is why Mo intakes are so high concerning reference values. Regarding Zn, intakes are similar to those reported by Wardenaar et al. [47] in 553 German elite athletes and lower than those reported in male swimmers [48]. High carbohydrate intake by soccer players may lead to a suboptimal Zn intake causing fatigue and decreased performance [49]. Lukaski et al. [50] reported that Zn nutritional status, among other MTEs, could be a predictor of physical performance in athletes. 

The present study showed that male soccer players had higher plasma and urine concentrations of Mo than female soccer players with no significant differences in intracellular compartments. Otag et al. [51] reported that serum Mo concentrations increased after performing 2 km on an ergometer. Similarly, Berger et al. [52] reported increased concentrations after a marathon. On the other hand, Maynar et al. [8] did not observe significant changes in serum Mo concentrations after an aerobic training period of 6 months. However, they observed that athletes had higher serum concentrations than the control group. When analyzing basal states, Maynar et al. [7,21] reported that athletes showed higher serum concentrations than the control group, with anaerobic athletes having the highest concentrations within the athlete group. In contrast, mid-level and high-level athletes had lower intracellular Mo concentrations than the control group.

The high concentrations of Mo in plasma and urine in male soccer players could facilitate the formation of uric acid, avoiding the damage of free radicals (superoxide anions) generated by xanthine oxidase in the ischemia–reperfusion processes generated during high-intensity physical exercise [53]. In relation to the above, uric acid concentrations appear to be higher in active men [54] and male athletes [55]. In addition, women seem to be less susceptible to oxidative stress, particularly premenopausal women, due to the antioxidant role of estrogen [56].

Regarding the results found in Zn, male soccer players showed higher Zn concentrations in plasma and urine. On the other hand, female soccer players showed relatively higher Zn concentrations in erythrocytes. Maynar et al. [7] showed that male athletes with high and medium levels of training had lower Zn concentrations than a control group of men. In women, Deuster et al. [57] observed no differences between high-level trained and untrained athletes in plasma and erythrocyte Zn concentrations. However, women with high-level training excreted a greater amount of Zn. When comparing between sport modalities, Nuviala et al. [58] reported that karatekas and runners had higher serum Zn concentrations. However, when urinary Zn excretion was analyzed, there were no differences. In erythrocytes, Singh et al. [59] reported higher Zn concentrations in female runners than female non-runners. When comparing sexes, Bordin et al. [60] showed higher plasma Zn concentrations in men, which were also higher after a treadmill test. Furthermore, Tipton et al. [61] observed that, under hyperthermia and normothermia conditions, men had significantly higher Zn losses in sweat compared to women. In soccer players, recently Toro-Román et al. [13] reported higher plasma and lower erythrocyte concentrations of Zn in male soccer players compared to a control group. However, Metin et al. [24] reported no differences in plasma Zn concentrations between groups, similar to those reported by the previous authors. No differences were observed either when comparing the time of match play in plasma Zn concentrations [23].

The differences in Zn concentrations in the various compartments analyzed could be due to different reasons. First, the difference in muscle mass between sexes. As discussed above, most Zn is found in skeletal muscle (~60%), whereas only ~0.1% of body Zn is found in plasma [62]. Secondly, this is due to the muscle damage produced during soccer practice generated by the predominant physical actions (accelerations, decelerations, changes of direction) and trauma due to collisions and impact [63,64]. It is known that there are differences between genders in the physical demands during a match, with more actions of greater intensity in men’s matches [65]. Muscle damage would lead to a greater release of Zn from the muscle into the plasma [27,66], and an increase in urinary excretion of Zn [67]. Due to the difference in muscle mass between sexes, markers of muscle damage are higher in men. Therefore, correlations are higher in male soccer players. Finally, the hemolysis produced by the predominant actions in soccer, mentioned above, could also explain the differences observed in the present study. In erythrocytes, Zn is found in the membrane and is a cofactor of CA and SOD [28]. As in the present study, Mundie et al. [27] observed after strength training, significant decreases in erythrocyte Zn concentrations together with a significant increase in plasma Zn. It is known that hemolysis could lead to increases in serum and plasma [68,69] since the concentration of Zn in erythrocytes is approximately 10 times higher compared to plasma or serum [70]. It should be noted that expressing intracellular TME concentrations in values relative to cells is important because, as in the present study, the results could be different. Although there were no differences in absolute Zn concentrations in erythrocytes between sexes, differences were observed when the concentrations were expressed in relation to the number of erythrocytes.

This research has a number of limitations: (a) a cross-sectional study was carried out at a specific time during the sport season; the time they had been training was not taken into account, (b) it would be interesting to add complementary Mo data such as molybenzymes, (c) the sample calculation was not performed; (d) the technical error of measurement of anthropometric parameters was not calculated; (e) muscle mass was not directly estimated using validated prediction models and (f) food consumption analyses were not performed by nutrition/dietetics professionals skilled in converting data obtained from questionnaires to continuous variables.

## 5. Conclusions

Mo and Zn concentrations differ between sexes in soccer players. Specifically, male soccer players showed higher extracellular (plasma and urine) Mo and Zn concentrations. However, female soccer players showed higher Zn concentrations relative to the number of erythrocytes.

There could be direct relationships between markers of muscle damage (CK and LDH) and muscle mass with extracellular (plasma and urine) Zn concentrations.

Differences in Mo concentrations could be due to higher uric acid values in men. On the other hand, discrepancies in Zn concentrations could be due to increased muscle damage, hemolysis, or differences in muscle mass.

## Figures and Tables

**Table 1:** General characteristics of the sample and of the training session carried out.

Parameters		Male Soccer Players (n = 68)	Female Soccer Players (n = 70)
Age (years)		20.61 ± 2.66	23.37 ± 3.95
Experience (years)		14.73 ± 3.13	14.51 ± 4.94
Playing position (%)	Goalkeeper	7.70	11.10
	Defense	30.80	33.30
	Midfielder	38.50	29.60
	Forward	23.10	25.90
Training (nº)		10.3 ± 2.4	11 ± 3.1
Training (min)		941.1 ± 75.6	1106.6 ± 126.3

**Table 2:** Anthropometry, body composition, physical condition and physical activity of the participants.

Parameters	Male Soccer Players (n = 68)	Female Soccer Players (n = 70)
Height (m)	1.76 ± 0.061	1.65 ± 0.06 **
Weight (kg)	71.50 ± 5.93	59.58 ± 7.17 **
Σ6 skinfold (mm)	60.34 ± 12.35	94.62 ± 18.54 **
Fat (%)	9.46 ± 1.30	18.16 ± 2.74 **
Muscle (%)	50.80 ± 1.67	45.62 ± 3.03 **
Bone (%)	15.71 ± 1.16	15.03 ± 1.55 *
Muscle/Bone	2.31 ± 0.24	1.86 ± 0.38 **
SJ (cm)	50.52 ± 6.48	35.65 ± 5.82 **
SJ (cm/kg)	1.39 ± 0.25	1.53 ± 0.41 *
SJ (s)	0.641 ± 0.041	0.539 ± 0.045 **
Isometric lower limb strength (kg)	137.47 ± 25.60	92.78 ± 23.17 **
Total time (min)	12.41 ± 1.58	9.18 ± 1.12 **
Speed (km/h)	19.17 ± 1.72	15.73 ± 1.16 **
VO_2max_ (mL/min/kg)	52.21 ± 2.91	39.72 ± 6.22 **
VCO_2max_ (L/min)	4.05 ± 0.36	2.68 ± 0.44 **
HR_max_ (bpm)	187.78 ± 6.52	183.33 ± 7.34
Physical activity (MET min/week)	5846 ± 2117	6271 ± 3201

* *p* < 0.05 differences in male vs. female soccer players; ** *p* < 0.01 differences male vs. female soccer players; Σ: summatory; SJ: squat jump; HR: heart rate; VO_2max_: maximal oxygen consumption; VCO_2max_: maximal carbon dioxide output.

**Table 3:** Energy, water, Mo and Zn intake of participants.

Parameters	Male Soccer Players (n = 68)	Female Soccer Players (n = 70)
Energy (kcal)	1796.0 ± 420.1	1531.3 ± 521.6 *
Water (L)	764.3 ± 226.5	791.2 ± 185.2
Mo (µg/day)	240.9 ± 99.1	251.8 ± 85.3
Zn (mg/day)	10.7 ± 3.1	9.5 ± 2.6

* *p* < 0.05 differences male vs. female soccer players; Mo: molybdenum; Zn: zinc.

**Table 4:** Hematologic and biochemical values of the participants.

Parameters	Male Soccer Players (n = 68)	Female Soccer Players (n = 70)
Erythrocytes (millions)	4.90 ± 0.32	4.31 ± 0.27 **
Platelets (thousands)	190.72 ± 48.21	206.98 ± 35.28 *
CK (U/L)	355.51 ± 187.01	158.54 ± 123.75 **
LDH (U/I)	515.46 ± 30.33	481.49 ± 24.59 **

* *p* < 0.05 differences male vs. female soccer players; ** *p* < 0.01 differences male vs. female soccer players.

**Table 5:** Mo concentrations in the different biological matrices analyzed.

Parameters	Male Soccer Players (n = 68)	Female Soccer Players (n = 70)
Plasma (µg/L)	2.49 ± 0.73	1.95 ± 0.71 **
Urine (µg/L)	53.76 ± 39.62	39.62 ± 33.63 *
Erythrocytes (µg/L)	27.29 ± 25.40	34.63 ± 31.51
Erythrocytes (pg/cell^−6^)	7.11 ± 6.34	7.93 ± 7.11
Platelets (µg/L)	15.55 ± 14.61	18.34 ± 15.85
Platelets (pg/cell^−3^)	0.0799 ± 0.0531	0.09211 ± 0.0834

* *p* < 0.05 differences male vs. female soccer players; ** *p* < 0.01 differences male vs. female soccer players.

**Table 6:** Zn concentrations in extracellular and intracellular biological matrices.

Parameters	Male Soccer Players (n = 68)	Female Soccer Players (n = 70)
Plasma (µg/L)	973.24 ± 167.18	837.92 ± 161.86 **
Urine (µg/L)	923.63 ± 459.74	551.56 ± 310.08 **
Erythrocytes (mg/L)	10.62 ± 1.57	10.80 ± 1.97
Erythrocytes (µg/cell^−6^)	2.21 ± 0.34	2.60 ± 0.48 **
Platelets (µg/L)	244.68 ± 77.57	229.70 ± 83.23
Platelets (pg/cell^−3^)	1.28 ± 0.46	1.18 ± 0.44

** *p* < 0.01 differences male vs. female soccer players.

**Table 7:** Correlations between extracellular Zn concentration with biomarkers of muscle damage (CK and LDH) and muscle percentage without differentiating between sexes.

Parameters	Soccer Players (n = 138)	
Zn Plasma (µg/L)	*r*	*p*
CK (U/L)	0.156	0.126
LDH (U/I)	0.137	0.205
Muscle (%)	0.297	0.001
Muscle/bone	0.201	0.027
Zn urine (µg/L)	*r*	*p*
CK (U/L)	0.269	0.002
LDH (U/I)	0.224	0.009
Muscle (%)	0.338	<0.001
Muscle/Bone	0.192	0.032

CK: creatine kinase; LDH: lactate dehydrogenase; *r*: Pearson’s correlation coefficient.

**Table 8:** Correlations between intracellular and extracellular Zn concentrations with biomarkers of muscle damage and muscle percentage according to sex.

Parameters	Male Soccer Players (n = 68)		Female Soccer Players (n = 70)	
Zn Plasma (µg/L)	*r*	*p*	*r*	*p*
CK (U/L)	0.170	0.160	0.129	0.322
LDH (U/I)	0.210	0.095	0.114	0.412
Muscle (%)	0.184	0.142	0.156	0.216
Muscle/bone	0.041	0.763	−0.168	0.182
Zn Urine (µg/L)	*r*	*p*	*r*	*p*
CK (U/L)	0.105	0.396	0.050	0.690
LDH (U/I)	0.194	0.116	0.120	0.352
Muscle (%)	0.103	0.425	0.152	0.223
Muscle/bone	0.154	0.213	0.080	0.524

CK: creatine kinase; LDH: lactate dehydrogenase; *r*: Pearson’s correlation coefficient.

## Data Availability

Not applicable.
